# Cognitive and functional dementia assessment tools: review of Brazilian literature

**DOI:** 10.1590/S1980-57642008DN10100004

**Published:** 2007

**Authors:** Luciano Góis Vasconcelos, Sonia Maria Dozzi Brucki, Orlando Francisco Amodeo Bueno

**Affiliations:** 1 Post-graduate student; From the Department of Psychobiology, Federal University of São Paulo, São Paulo, Brazil.; 2 Affiliated researcher; From the Department of Psychobiology, Federal University of São Paulo, São Paulo, Brazil.; 3 Adjunct Professor and Head of Department. From the Department of Psychobiology, Federal University of São Paulo, São Paulo, Brazil.

**Keywords:** neuropsychological evaluation, functional evaluation, dementia, neuropsychology, demência, avaliação cognitiva, avaliação funcional, neuropsicologia

## Abstract

The diagnosis of dementia is based on cognitive and functional evaluation. One of the difficulties in ascertaining the number of people with dementia in developing countries is the population’s lack of formal education. Independent effects of age, sex and education have been identified on scores for most but not all cognitive tests.

**Objectives:**

Identify the most-used cognitive and functional assessment tools in Brazil, related to dementia diagnosis and treatment outcome; and identify adaptations or normative data, when available.

**Methods:**

Data were generated from PubMed, LILACS and Portal Periodicos CAPES (thesis database) databases using the search terms ‘dementia’ and ‘Alzheimer’. Data collection criteria were a. Articles with abstract; b. Brazilian abstracts, related to adult Brazilian population; c. Clear mention of assessment tool in the abstract text. A total of 108 abstracts were selected for the main analysis: a. to identify the instruments used b. to determine how many of the selected abstracts mentioned each tool and c. to search in the mentioned databases for respective test adaptations or normative data.

**Results:**

Some 52 different assessment tools, 41 cognitive instruments and 11 functional instruments were identified. The most cited assessment tests were the Mini Mental State Examination (64 abstracts) and Pfeffer Functional Activities Questionnaire (4 abstracts).

**Discussion:**

Many of the instruments used only have the description of the translation process into Portuguese, along with some suggestions of validation or normative data. Few of these followed the recommended procedures of validation, replication, normalization or transcultural adaptation.

## Abstract (pt)

O diagnóstico de demência é baseado na avaliação cognitiva e funcional. Países em desenvolvimento deparam-se com uma dificuldade adicional, relacionada à baixa escolaridade formal da população. Idade, escolaridade e sexo exercem indiretamente efeitos sobre os escores da maioria dos testes cognitivos.

**Objetivos:**

Identificar os testes cognitivos e funcionais mais utilizados no Brasil, relacionados à avaliação diagnóstica e resultado de tratamento de demências; e identificar adaptações ou normatizações, quando disponíveis.

**Métodos:**

As informações foram obtidas dos bancos de dados PubMed, LILACS e Portal Periódicos CAPES (banco de teses). Digitou-se as palavras-chave ‘dementia’ e ‘Alzheimer’. Os resumos analisados foram selecionados da pesquisa inicial através dos seguintes critérios: a. artigo com resumo; b. resumos brasileiros, relacionados à população adulta brasileira; c. clara menção do teste utilizado no texto do resumo. Os 108 resumos selecionados foram submetidos à seguinte avaliação: a. identificar os instrumentos utilizados; b. determinar o número de citações de cada teste nos diferentes resumos; c. identificar adaptações e normatizações dos testes nos bancos de dados avaliados.

**Resultados:**

foram identificados 52 diferentes instrumentos, 41 testes cognitivos e 11 funcionais. Respectivamente, os mais citados foram o Mini Exame do Estado Mental (64 resumos) e o Questionário de atividades funcionais de Pfeffer (4 resumos).

**Discussão:**

A maioria dos instrumentos utilizados tem apenas a descrição do processo de tradução, algumas sugestões de validação ou normatização. Poucos trabalhos desenvolveram todos os procedimentos recomendados para validação, replicação, normatização e adaptação trans-cultural.

## Global aging trends

The aging of the world's population is the result of two factors: declines in fertility and increases in life expectancy. Fertility rates have declined in developing countries over the past 30 years. In addition, in developed countries, the largest gain ever in life expectancy at birth occurred during the 20^th^ century, averaging 71% for females and 66% for males. Life expectancy at birth in developed countries now ranges from 76 to 80 years.

In 2000, the worldwide population of persons aged ≥65 years was an estimated 420 million. During 2000–2030, the worldwide population aged ≥65 years is projected to increase by approximately 550 million to 973 million, increasing from 6.9% to 12.0% worldwide, from 15.5% to 24.3% in Europe, from 12.6% to 20.3% in North America, from 6.0% to 12.0% in Asia, and from 5.5% to 11.6% in Latin America and the Caribbean. Between 2000–2030, the number of persons in developing countries aged ≥65 years is projected to almost triple, from approximately 249 million in 2000 to an estimated 690 million in 2030, and the developing countries' share of the world's population aged ≥65 years is projected to increase from 59% to 71%^1^.

## Brazilian aging trends

The Brazilian elderly population (≥60 years) increased from 3 million in 1960 to 14 million by 2002. It is projected to reach 32 million by 2020^2^.

Compared with developed countries, the Brazilian aging process has been faster, had deeper structural changes and has also taken place in a younger population^3^.

## Dementia and aging

Several authors have highlighted that dementia is one of the chronic conditions that will affect a considerably increased number of elderly both in developed and developing countries.

A large number of epidemiological surveys show very similar results such as increasing rates with rising age^4^.

As adults live longer, the prevalence of Alzheimer's disease, which doubles every 5 years after age 65, is also expected to increase. Approximately 10% of adults aged ≥65 years and 47% of adults aged ≥85 years suffer from this degenerative and debilitating disease^5^.

## Dementia around the world

The worldwide number of persons with dementia in 2000 was estimated at about 25 million persons. Almost half of the demented persons (46%) lived in Asia, 30% in Europe, and 12% in North America. Fifty-two percent lived in less developed regions. About 6.1% of the population 65 years of age and older suffered from dementia (about 0.5% of the worldwide population) and 59% were female. The number of new cases of dementia in 2000 was estimated to be 4.6 million (one new case every 7 seconds). The forecast indicated a considerable increase in the number of demented elderly from 25 million in the year 2000 to 63 million in 2030 (41 million in less developed regions) and to 114 million in 2050 (84 million in less developed regions)^6,7^.

Dementia has already been established as one of the major health challenges of this century due to the enormous burden these pathologies impose on health care systems. Dementia is a significant public health problem as it is one of the most common diseases in the elderly and a major cause of disability and mortality (Ritchie and Lovestone, 2002)^8^. Brazilian dementia prevalence has been estimated at 7.1% (54.1% Alzheimer Disease)^9^.

## Dementia assessment tools

The dementia diagnosis is based on cognitive and functional evaluation. Some diagnostics criteria (DSMIII, DSM-IV) highlight instrumental cognitive domain evaluations.

Use of validated assessment tools provides structure for the assessment process, helps assure consistency, and provides a mechanism for periodic re-evaluation. The assessment approaches also foster a common language for the health care team and consist of measurable parameters that can be used to monitor outcomes^10^.

Physicians often underestimate the extent of disability that a patient has in basic ADLs. Further, physicians’ recording of the level of function in medical notes is poor. By using standardized assessment tools, the evaluation can objectively document physical, cognitive, emotional, and functional conditions. Based on the evidence, assessment tools should be chosen to aid in diagnosis and to measure outcome treatment of dementia. Older patients should be screened by standardized assessment tools in order to improve diagnosis, assessment, and outcome measurement. There are many commonly used tools in dementia assessment^11^.

## Cross-cultural adaptation, validation and reliability

One of the difficulties in ascertaining the number of people with dementia in developing countries is that many older people in these regions have little if any formal education, and often cannot read or write^12^.

Independent effects of age, sex, education and occupation were identified on scores for most but not all cognitive tests^13^. Considering that Brazil has a high number of illiterates and low educational subjects, such instruments should be adapted^14^.

## Objectives

- To identify the most-used cognitive and functional assessment tools in Brazil, related to dementia diagnosis and treatment outcome;
- To identify adaptations or normative data, when available.

## Methods

The literature search was performed up until September 2006. Data were generated from PubMed, LILACS and Portal Periodicos CAPES / Brazilian thesis and dissertations database (PPC) databases using the search terms ‘dementia’ and ‘alzheimer’. For the PubMed search the term “brazil” was also included. The LILACS search only considered abstracts in English or Portuguese.

Data collection criteria: a. Articles with abstract; b. Brazilian abstracts, related to adult Brazilian population; c. Clear mention of assessment tool in the abstract text.

The search result of three databases (Table 1) was submitted to criteria above and the repeated abstracts were excluded. 108 abstracts were selected for the following analysis: a. to identify the instruments used; b. to determine how many of the selected abstracts cited each tool; and c. to search in the mentioned databases for respective test adaptations or normative data.

**Table 1:** Database search results.

Database	Search results
Pubmed	98
LILACS	456
PPC^†^	284
Partial result	838
Final result*	108

* After exclusion of repeated abstracts and submission to criteria collection.

† Portal periódicos CAPES/Brazilian Thesis and Dissertations Database.

## Results

A total of 52 different assessment tools were identified: 41 cognitive instruments and 11 functional instruments.

The most mentioned assessment tests were the Mini Mental State Examination (64 different abstracts) and the Pfeffer Functional Activities Questionnaire (4 different abstracts). All the mentioned instruments and respective suggestions of adaptations or normative data can be found in Tables 2 and 3.

**Table 2:** The most used cognitive assessment tools in Brazil.

	Abstracts	Adaptations or
Cognitive assessment tool	citation*	normative data†
1. Mini Mental State Examination	64	15
2. Verbal Fluency	9	16
3. Trail Making	8	17,18
4. Digit Span	8	17,18
5. Blessed's Information-Memory-Concentration Test	7	NS ‡
6. CAMDEX / CAMCOG	7	19
7. Wechsler Adult Intelligence Scale - III	6	20
8. Boston Naming Test	5	21, 22
9. Neuropsi	5	23
10. Clock Drawing Test	5	24
11. Alzheimer's Disease Assessment Scale (ADAS-Cog)	4	25
12. Wechsler Memory Scale-Revised	4	26
13. Informant Questionnaire On Cognitive Decline In The Elderly (IQCODE)	3	NS
14. Consortium To Establish For Alzheimer Disease Battery (CERAD)	3	27
15. Clinical Interview Schedule	3	28
16. Wisconsin Card Sorting Test	3	29
17. Buschke Selective Reminding Test	3	30
18. Brazilian Version Of The Mattis Dementia Rating Scale (DRS)	3	31, 32
19. Computerized Neuropsychol Test Battery (CNTB)	2	33
20. Stroop Test	2	34
21. Cognitive Abilities Screening Instrument - Short Form (CASI-S)	2	35
22. Protocole D'évaluation Neuropsycol Optimal Du Montreal	2	36
23. FAS Verbal Fluency	2	37
24. Word Span	2	38
25. Rey Auditory Verbal Learning Test	2	39
26. Objects Presented As Simple Drawings	1	40,17
27. Spatial Recognition Span	1	38
28. Brief Cognitive Screening Battery (BCSB)	1	NS
29. SIDAM Portuguese Version	1	41
30. Reduced Version Of The Face-Hand Test	1	42
31. Benton Visual Recognition Test	1	NS
32. Token Test	1	43
33. International Affective Picture System (IAPS)	1	44
34. Spatial Recognition Span	1	39
35. Porteus Mazes Test	1	NS
36. Bell Test	1	NS
37. Luria's Fist-Edge-Palm Test	1	45
38. California Verbal Learning Test	1	NS
39. Set-Test	1	NS
40. Short Cognitive Performance Test	1	46
41. Fuld Object Memory Evaluation (FOME)	1	NS

* number of abstracts citing the instrument;

† reference number of adaptation or normative data suggestion;

‡ NS, no suggestions.

**Table 3:** The most used functional assessment tools in Brazil.

	Abstracts	Adaptations or
Functional assessment tools	citation*	normative data†
1. Pfeffer Functional Activities Questionnaire	4	NS ‡
2. Activities of Daily Living (ADL) + Instrumental Activities of Daily Living (IADL)	3	47
3. Activities of Daily Living (ADL)	3	48
4. Hoehn & Yahr Staging	2	49
5. Schwab & England Scale (SES)	2	NS
6. Katz' Index of ADL	2	NS
7. Functional Assessment Staging (FAST)	2	NS
8. Bayer-Activities of Daily Living	2	NS
9. Unified Parkinson's Disease Rating Scale-Activities of Daily Living Section (UPDRS-ADL)	1	NS
10. Barthel Index	1	50
11. Activities of Daily Living - International Scale	1	NS

* number of abstracts citing the instrument;

† reference number of adaptation or normative data suggestion;

‡ NS, no suggestions.

Many of them only describe the translation process to Portuguese, with some superficial suggestions of validation, replication, normative data or trans-cultural adaptation. Few of them developed the recommended procedures outlined earlier.

## Discussion

Many Brazilian authors have shown the influence of education on test scores: the Mini Mental State Exam-ination^15^, Verbal Fluency Test^16^, Boston Naming Test^21,22^, ADAS-Cog^25^, CERAD^26^,Mattis Dementia Rating Scale^30,31^.

Several dementia assessment tools were identified, where few of these have followed the recommended procedures of validation, replication, normative data or trans-cultural adaptation. In the context of research use this could be acceptable, but these procedures are absolutely necessary for epidemiological surveys.

Psychometric properties of scales and trans-cultural adaptations should be developed to minimize educational influence and decrease false positive diagnosis for cognitive impairment.

Health-care professionals should be trained to recognize and evaluate quality scales and psychometric concepts such as reliability (internal consistency and testretest), validity (construct, content, face and criterion validities), and sensitivity to change (responsiveness).

Methodological problems need to be addressed, particularly development of culture- and education-fair dementia diagnostic procedures.

Consensus on assessment and outcome tools would facilitate multi-center comparative studies. One method of achieving these research goals would be through a consensus conference.

Future research should emphasize functional State, quality of life, and caregiver burden, as well as economic factors and societal perspectives.
